# Treatment of Phlegmonous Inflammations

**Published:** 1895-11

**Authors:** 


### Treatment of Phlegmonous Inflammations.

In the lesser phlegmonous inflammations alcohol dressings abort the inflammatory process and in the graver forms they cause the rapid formation of a circumscribed abscess filled with thick pus. Alcohol of 60 to 90 degrees is to be used. The skin of the inflamed area is cleansed with ether; if there is any open wound it is covered with powdered iodoform; then a thick, even layer of cotton soaked with alcohol is applied with a proper protective over it; this latter is pierced with holes or slits so as to allow evaporation of the alcohol and prevent any caustic action. The dressing is kept in place for twenty-four hours and must be renewed daily for some days after the disappearance of all tumefaction.—*Gaz. Med. de Paris, No.* 28, 1895.

